# Water Management Interventions, Organic Fertilization, and Harvest Time in Dry Land in the Biosaline Production of Cactus Pear

**DOI:** 10.3390/plants13182540

**Published:** 2024-09-10

**Authors:** Tarcia Carielle Miranda Dantas Nunes, Gherman Garcia Leal de Araújo, Thieres George Freire da Silva, Tadeu Vinhas Voltolini, Glayciane Costa Gois, Cleyton de Almeida Araújo, Anderson de Moura Zanine, Daniele de Jesus Ferreira, Danillo Marte Pereira, Francisco Naysson de Sousa Santos, Henrique Nunes Parente, Silvia Helena Nogueira Turco, Michelle de Oliveira Maia Parente, Fleming Sena Campos

**Affiliations:** 1 Department of Animal Science, Center for Agricultural Science Universidade Federal do Vale do São Francisco, Petrolina 56300-990, PE, Brazil; tccarielle@hotmail.com; 2 Department of Animal Production, Embrapa Semiárido, Empresa Brasileira de Pesquisa Agropecuária, Petrolina 56302-970, PE, Brazil; gherman.araujo@embrapa.br (G.G.L.d.A.); tadeu.voltolini@embrapa.br (T.V.V.); 3 Academic Unit of Serra Talhada, Universidade Federal Rural de Pernambuco, Serra Talhada 56909-535, PE, Brazil; thieres.silva@ufrpe.br; 4 Department of Animal Science, Universidade Federal do Maranhão, Chapadinha, Maranhão 65500-000, MA, Brazil; glayciane_gois@yahoo.com.br (G.C.G.); anderson.zanine@ufma.br (A.d.M.Z.); daniele.ferreira@ufma.br (D.d.J.F.); nayssonzootecnista@gmail.com (F.N.d.S.S.); hnparente@hotmail.com (H.N.P.); 5 Department of Animal Science and Pasture, Universidade Federal do Agreste de Pernambuco, Garanhuns 55292-270, PE, Brazil; alcleytonaraujo@hotmail.com; 6 Department of Animal Science, Universidade Federal da Paraíba, Areia 58051-900, PB, Brazil; danillomarter.zootec@gmail.com; 7 Postgraduate Program in Agricultural Engineering, Universidade Federal do Vale do São Francisco, Petrolina 56300-990, PE, Brazil; shnturco@gmail.com; 8 Postgraduate Program in Tropical Animal Science, Universidade Federal do Piauí, Teresina 64049-550, PI, Brazil; michellemrn14@gmail.com

**Keywords:** biosaline agriculture, brackish water, *Opuntia strict* (HAW.)

## Abstract

Brackish water can promote physicochemical changes in the soil. Aiming to mitigate the effect of excess salts in the soil, the use of organic matter promotes restructuring. The aim was to evaluate the productive and nutritional characteristics of cactus pear under different brackish water depths (ID) and levels of organic matter (OM). A factorial arrangement of 4 × 4 × 4 with four replications was utilized. Plots consisted of ID (0, 12, 20, and 28% reference evapotranspiration—ETo), and subplots were composed of OM levels (0, 15, 30, and 45 t/ha) and days after planting (DAP; 180, 270, 360, and 450 days). The growth, yield, and chemical composition of cactus pear were affected by ID and OM and/or by their interaction. The regular and increasing application of ID from 192 to 456 mm/year and a rainfall of 110 mm/year in cactus pear crops in biosaline systems improves the growth, freshness, dry matter yields, accumulation capacity per unit area, and chemical composition of cactus pear. The increase in OM up to the range from 30 to 45 Mg/ha linearly increases the agronomic performance of cactus pear. Biosaline systems with cactus pear should be adopted with the combined use of regular supplementary ID and OM, measuring at 304 mm/year and 45 Mg/ha, respectively.

## 1. Introduction

Arid and semiarid lands are strongly influenced by a negative water balance resulting from irregular and scarce rainfall, in addition to high temperatures and high evaporation. Due to these characteristics, water sources are considered insufficient in these regions [1]. Thus, it is common to use water from wells that are often not suitable for irrigation, contributing to the accumulation of salts in the soil. In this situation, alternative drought- and salinity-tolerant crops that can survive in adverse conditions are crucial for sustainability in semiarid regions [2,3].

Biosaline agriculture emerges as a sustainable alternative. This practice consists of growing plants using water with high levels of salts in environments that already suffer from salinity problems [4]. In line with this approach, studies seek to evaluate the efficiency of using brackish water in crop irrigation, especially in regions where potable water is scarce and limited. This is the case of Brazilian semiarid regions, where brackish water is used for irrigation in crops tolerant to high salt content or native vegetation [5]. Cactus pear has great importance in semiarid regions [6]. Its physiology is characterized by a carbon fixation pathway called Crassulacean acid metabolism (CAM), in which there is a reduction in water loss due to diurnal stomatal closure, nocturnal stomatal opening, and CO_2_ fixation [7]. As a forage, cactus pear has high digestibility, a high content of soluble carbohydrates (523–555 g/kg dry matter) [8], and water accumulation in cladodes (928.8 g/kg natural matter) [9], which guarantees a water supply for animals. However, the low content of crude protein (44.6 g/kg dry matter) [8], neutral detergent fiber (260.3 g/kg dry matter) [9], and acid detergent fiber (146 g/kg dry matter) [8] impede its supply as the sole source of feed to animals.

Despite the high efficiency in the use of water from cactus pear, the low rainfall and high temperatures of the semiarid region reduce its productivity. Thus, the application of saline water through irrigation in a biosaline farming system becomes an alternative to maximize cactus pear productivity, while excessive brackish water depths intensify the load of salts in the soil [10] and, depending on the exposure time and the level of salinity, the plant can suffer a toxic effect due to the excess of absorbed salts, causing a drop in the yield [11,12].

Plant tolerance to salinity is dependent on different physiological interactions [13]. At the same time, the accumulation of specific ions inside the plant, such as sodium, chloride, and boron, can directly interfere with internal biochemical processes [14]. However, research has shown that irrigation with brackish water for short periods does not significantly reduce crop productivity [15], requiring more detailed studies on the reality of the Brazilian semiarid region, seeking ways that promote better coexistence with conditions of water scarcity [16].

Efficient and low-cost strategies to optimize the use of brackish water in semiarid environments are needed to reduce soil and plant toxicity [15]. The application of organic matter to mitigate the effects of salinity on plants is a strategy to increase the availability of nutrients for plants and stimulate their development, increasing crop productivity [1,17,18]. The attenuation of harmful impacts can be attributed to the presence of humic substances contained in organic inputs that provide greater osmotic regulation between the root and the soil solution and enable increased crop productivity [18,19], in addition to its importance in sustainable land use and agricultural productivity.

The yield potential of cactus pear under irrigation conditions with saline water in the Brazilian semiarid region is still little known [20,21]. In addition, studies that indicate the amount of brackish water and the ideal dose of organic matter that can favor the production of cactus pear biomass are incipient, and research is needed to support this management strategy. Thus, the hypothesis of our work is that the use of organic matter loads improves the growth, productivity, and chemical composition of cactus pear and reduces the negative impacts of irrigation with brackish water in semiarid conditions. The objective was to evaluate the productive and nutritional characteristics of cactus pear under different brackish water depths and levels of organic matter in a biosaline agriculture system in a Brazilian semiarid region.

## 2. Materials and Methods

### 2.1. Experiment Location

The study was carried out in the area of Prospecting in Biosaline Agriculture Studies of the Caatinga Experimental Field. Rainfall is concentrated between the months of November and April, with the average annual rainfall being around 400 mm, irregularly distributed.

**Figure 1:**
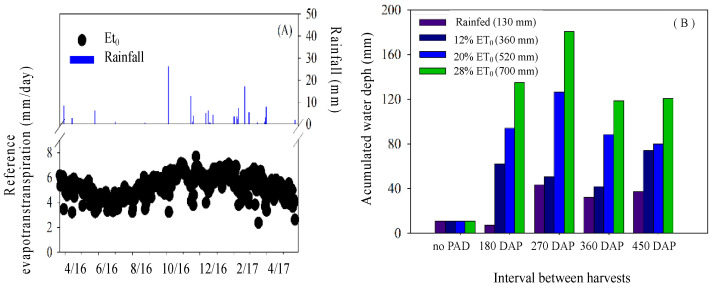
Reference evapotranspiration and daily rainfall (**A**), and cumulative irrigation brackish water depths (**B**) applied during the period of the absence of differentiation (PAD) (3 M duration), between differentiation and the first sampling of 180 DAP, and from 270, 360, and 450 days after the planting (DAP) of cactus pear in the biosaline agriculture system in the municipality of Petrolina, state of Pernambuco.

The experimental period lasted 450 DAP (days after planting). During the experimental period, reference evapotranspiration was 2237 mm (equivalent to 1890 mm/year) and rainfall was 131 mm (equivalent to 104 mm/year) (Figure 1). The cactus used in the experiment was the Mexican Elephant Ear (*Opuntia stricta* Haw.). The cladodes came from the experimental field of the Brazilian Agricultural Research Corporation—Embrapa Semiarido—PE. Cladodes of plants eighteen months after planting were selected. After being cut, the cladodes were stored in a warehouse with open sides and covered with zinc tiles. The cladodes underwent a rest period of fifteen days before planting so that healing could occur. After this period, they were planted using one cladode per hole in a vertical position so that 50% of their surface was buried. Cactus pear was planted and kept under dry conditions for one year, until the establishment of the crop.

### 2.2. Soil, Water, and Organic Matter

The soil of the experimental area was classified as Plinthic Abrupt Eutrophic Yellow Argisol [22], situated on a flat relief, with medium texture. The soil chemical properties are listed in Table 1.

The soil chemical properties analyzed were as follows: pH through the addition of 25 mL deionized water in the soil sample and, after 30 min, the pH is measured with a previously calibrated potentiometer (Digimed, model dmph^−2^). The electrical conductivity (EC) was measured after the addition of deionized water until the formation of a saturation paste; after a rest period of 24 h, the paste was filtered through filter paper; and, subsequently, the EC was measured using a conductivity meter (AZ instrument Corp, model 6505, Taichung City, Taiwan) [23].

The concentration of total C and N was performed according to Bataglia et al. [24], through dry combustion (1000 °C) using an elemental analyzer (LECO Truspec CHNS, SDCHN636, Xiangtan City, China) calibrated with the wheat standard LECO 502–278 (C = 45.00% and N = 2.68%). Phosphorus (P), potassium (K^+^), and sodium (Na^+^) contents were determined with Mehlich^−1^ and quantified in a spectrophotometer (P) and flame photometer (K^+^ and Na^+^). Calcium (Ca^2+^) and magnesium (Mg^2+^) contents were extracted with KCl 1.0 mol L^−1^ and quantified in an atomic absorption spectrophotometer [25].

Exchangeable acidity (H + Al) was quantified by extraction with 0.5 M L^−1^ calcium acetate buffered at pH 7.0, which was subsequently titrated with 0.02 M L^−1^ NaOH solution [26]. Subsequently, the sum of bases (SB) was calculated using the equation SB = Ca + Mg + Na + K; the cation exchange capacity was calculated from the equation CEC = SB + H + Al, alongside the base saturation (V).

Irrigation was performed based on the Eto fraction according to the treatments (0, 12, 20, and 28%.Eto). Irrigation was performed three times a week using drip tapes containing emitters spaced at 0.20 m, with a flow rate of 0.9 L/h and a uniformity coefficient of 93%. Eto values were determined using the original Penman–Monteith equation [27]:

ETo=[0.408×Δ×(Rn – G)+γ×(900/Tm+273)×u2×(es – ea)]/[Δ+γ×(1+0.34×u2)]

where *ETo*—reference evapotranspiration; *Rn*—radiation balance at the crop surface; *G*—soil heat flux density; *Tm*—air temperature at the height of 2 m; *u*2—wind speed at 2 m height; *es*—saturation vapor pressure; *ea*—partial vapor pressure; Δ—the slope of the saturation vapor pressure curve; and *γ*—psychrometric coefficient. The meteorological data for determining Eto came from a meteorological station belonging to the National Institute of Meteorology [28], located near the experiment. The irrigation water came from underground wells, with an approximate flow rate of 1500 L/h.

Water samples were collected weekly for physical and chemical analyses. The water used in the experiment was classified as C3S1, i.e., with high salinity, low sodium content, and moderate hardness (75–150 mg/L) based on calcium carbonate [29]. The organic matter used was tanned bovine manure, containing the following characteristics: electrical conductivity = 2.20 mS/cm; pH = 8.1; carbon = 120 g/kg; nitrogen = 9.3 g/kg; calcium = 5.5 cmolc/dm^3^; magnesium = 5.9 cmolc/dm^3^.

### 2.3. Experimental Design

The cactus pear studied was the clone Orelha de Elefante Mexicana (*Opuntia stricta* Haw.). The experimental area (2720 m^2^) was implemented in 2015 with spacing of 1.6 × 0.4 m, totaling 15.625 plants/ha. Before planting, the soil in the experimental area was prepared through plowing, harrowing, and furrowing, and the cladodes were placed in the soil, with 50% of their length buried. Dry conditions were used for one year, until the establishment of the crop. Throughout the crop cycle, the necessary cultural treatments were carried out to reduce the incidence of spontaneous weeds and pests. The area was subjected to a uniform cut in March 2016, and the treatments were delimited. The experiment began in March 2016 and ended in March 2017.

This was a randomized block experimental design, in a factorial arrangement of 4 (irrigation depths) × 4 (levels of organic matter), with 4 replications, totaling 64 subplots. Each subplot contained 5 rows with 50 plants and a total area of 32 m^2^ (8 × 4 m), of which 15.36 m^2^ (4.8 × 3.2 m) refers to the useful area. Of the 50 plants, 26 plants near to the borders were excluded, leaving only 24 plants per subplot. 

The main plot consisted of three irrigation depths, namely 12%, 20%, and 28% Eto, where Eto is the reference evapotranspiration calculated according to [27], plus the rainfed condition, (control) which, when rainfall was added (130 mm, equivalent to 104 mm/year), resulted in 370 mm (240 + 130, equivalent to 296 mm/year), 520 mm (390 + 130, equivalent to 416 mm/year), and 700 mm (570 + 130, equivalent to 560 mm/year). The subplots were composed of four levels of organic matter (0, 15, 30, and 45 t/ha).

### 2.4. Growth, Productivity, and Chemical Composition of Cactus Pear

Data on the growth, productivity, and chemical composition of the crop were obtained at 180, 270, 360, and 450 days after planting (DAP) at the time of harvest.

For growth data, measurements were taken on two plants per subplot. With the aid of a measuring tape, the height (H, in cm) and the width of the plant (WP, in cm) were measured. The total number of cladodes (NC) per plant evaluated was also recorded.

A plant from the useful area of each subplot was sampled and weighed on a digital electronic scale (Toledo Prix, model 3400, 30 kg Capacity, São Paulo, SP, Brazil) to obtain the fresh weight per plant (FWP, kg/plant) and, then, the fresh matter productivity (FMP, t/ha) by the product between the FWP and the equivalent density of plants per hectare (DPH), considering the 15,625 plants/ha.

Three cladodes representing each subplot were sampled and weighed to obtain the individual cladode fresh mass (CFM, g/cladode). Then, cladodes were fragmented, homogenized, placed in trays, and taken to a forced ventilation oven (Tecnal, TE-394/2-MP, Piracicaba, SP, Brazil) at 55 °C until a constant weight to obtain the individual cladode dry mass (ICDM, g/cladode). The ICDM/CFM ratio resulted in the dry matter content (DMC, g/g). From the product of the FMP with the DMC, the dry matter productivity (DMP, t/ha) was obtained. With the difference between the DMC and DMP, the crop’s water accumulation (WA, kg/ha/mm) was estimated. Water-use efficiencies based on fresh (WUE-FM, kg/ha/mm) and dry (WUE-DM, kg/ha/mm) matter were determined according to Silva et al. [30].

Samples were ground in a knife mill (Wiley, Marconi, MA-580, Piracicaba, Brazil), using 1 mm sieves. Laboratory analyses were performed using the methods described by [23] for dry matter (DM; method 967.03), mineral matter (MM; method 942.05), crude protein (CP; method 981.10), and ether extract (EE; method 920.29). The neutral detergent fiber (*NDF*) and acid detergent fiber (*ADF*) content were determined as described by [31]. Lignin (*LIG*) was determined by treating acid detergent fiber residue with 72% sulfuric acid [32]. Hemicellulose (*HEM*) and cellulose (*CEL*) were calculated using the following equations:

(1) HEM=NDF – ADF

(2) CEL=ADF – Lignin

The total carbohydrates (*TCs*) were estimated using the equation proposed by [33]:

(3) TC=100 – (% CP+% EE+% MM)

The non-fiber carbohydrate (*NFC*) content was calculated as proposed by [34]:

(4) NFC=%TC−%NDF

### 2.5. Statistical Analysis

Data were subjected to Shapiro–Wilk and Levene tests to check for residual normality and variance homogeneity, respectively; and, once the assumptions were met, they were tested by analysis of variance (ANOVA) and, when there was an isolated effect of the factors brackish water depths and levels of organic matter, a regression analysis was performed. Data were analyzed using the Statistical Analysis System 9.1 (SAS Institute, Cary, NC, USA) [35]: Means were compared by Tukey’s test at a significance level of 5%.

## 3. Results

There was no interaction found among LA × AO × DAP for the biometric and biomass data of forage cactus. Interaction was observed only for dry matter and total carbohydrates values (*p* < 0.05) in the chemical composition (Table 1). Higher values for the percentage of dry matter were observed at 180 (128.4 g kg^−1^, *p* = 0.0004), 360 (107.7 g kg^−1^, *p* = 0.0008), and 450 mm (95,1 g kg^−1^, *p* < 0.001), associated with the lower LA levels without the use of organic fertilization (*p* < 0.05). There was an effect of LA × MO at 180 DAP on the total carbohydrates, with higher values at 450 DAP using 130 to 370 mm LA with 30 to 45 ton/ha of MO from 784.44 to 827.76 g kg^−1^ (*p* < 0.001), respectively.

An interaction between AO and LA was observed for plant height, the number of cladodes, green matter production, dry matter production, ad water accumulation. Higher values were found at higher levels of AO (30 and 45 ton/ha) and the higher LA (700 mm) compared to the lower LA (130 mm), with average values of 64.59 cm, 16.46 units, 112.04 t/ha, 8.80 t/ha, and 103.23 t/ha, respectively (Table 2).

An isolated effect of brackish water use and organic matter on water productivity, water-use efficiency, and plant width was observed (*p* < 0.05). The bromatological chemical composition was affected by brackish water irrigation (*p* < 0.05) for the variables neutral detergent fiber (NDF), acid detergent fiber (ADF), non-fibrous carbohydrates (NFC), hemicellulose, lignin, crude protein (CP), and mineral matter (MM). It was observed that the use of organic fertilization independently affected the levels of CP, ether extract (EE), hemicellulose, and lignin (*p* < 0.05).

**Figure 2:**
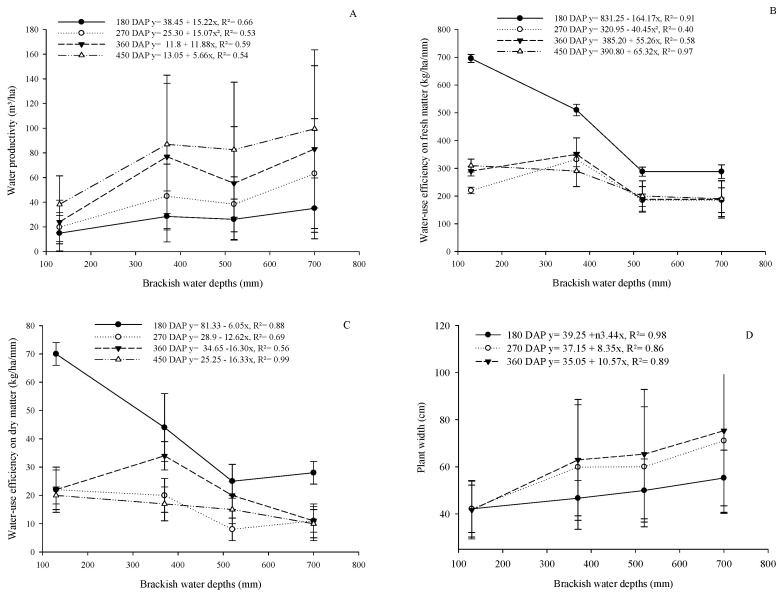
Effect of brackish water depths on the water productivity (**A**), water-use efficiency on fresh matter (**B**), and water-use efficiency on dry matter (**C**) and plant width (**D**) of cactus pear under different levels of organic matter in a biosaline agriculture system.

There was an increasing linear model adjustment (*p* < 0.05) of water accumulation (WA) as a function of the applied brackish water depths (Figure 2A). Adjusting the equation showed an increase in WA of 5.66; 11.88; 15.07; and 15.22 kg/ha/mm for every 1 mm of irrigated brackish water (Figure 2A), respectively, at 180; 270; 360; and 450 DAP.

The water-use efficiency based on fresh matter was influenced in a decreasing linear manner (*p* < 0.05) as a function of brackish water depths at 180; 270; 360; and 450 DAP (Figure 2B), with a reduction of 164.17; 40.45; 55.26; and 65.32 kg/ha/mm per 1 mm of irrigated brackish water depths (Figure 2B), respectively. Water-use efficiency based on dry matter was observed to be linearly decreasing as a function of water depth with a reduction rate of 72.84; 53.46; 47.58; and 56.87% when comparing the irrigation depth of 720 mm with that of 130 mm, respectively, at 180; 270; 360; and 450 DAP (Figure 2C). The brackish water depths increased (*p* < 0.05) the plant width linearly at 180; 270; and 360 DAP with increase rates of 24.64; 65.87; and 82.69% for the 700 mm brackish water depth compared to the 130 mm brackish water depth, respectively (Figure 2D).

**Figure 3:**
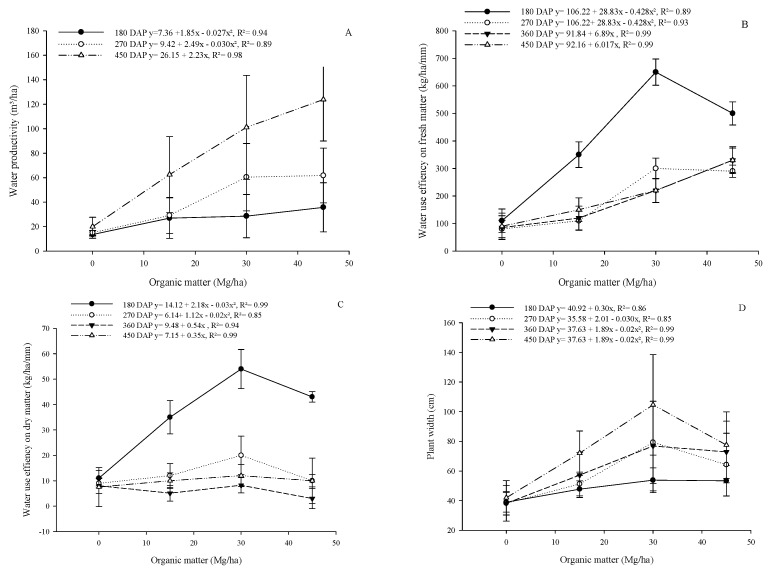
Effect of levels of organic matter on water productivity (**A**), water-use efficiency on dry matter (**B**), and water-use efficiency on fresh matter (**C**) and plant width (**D**) of cactus pear in a biosaline agriculture system.

There was a quadratic effect at 180 and 270 DAP, while at 450 DAP, it presented an increasing linear effect (*p* < 0.05; Figure 3A). There was no effect of organic matter levels on water production at 360 DAP (Figure 3A). Water-use efficiency on a dry matter basis and water-use efficiency on a fresh matter basis showed a quadratic effect at 180 and 270 DAP (Figure 3B,C). In relation to 360 and 450 DAP, there was an increasing linear effect (Figure 3B,C) for water-use efficiency on a dry matter and on a fresh matter basis. Plant width showed an increasing linear effect (*p* < 0.05) at 180 DAP with an increase of 0.30 cm for every 1 Mg/ha of organic matter (Figure 3D). There was a quadratic effect on the plant width at 270, 360, and 450 DAP (Figure 3D).

**Figure 4:**
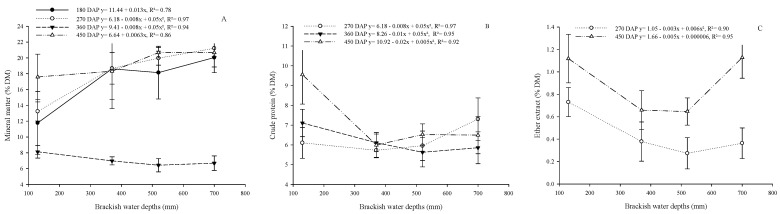
Effect of brackish water depths on the chemical composition of cactus pear under different doses of organic matter in a biosaline agriculture system. (Dry matter (**A**), mineral matter (**B**), crude protein (**C**). FM—fresh matter; DM—dry matter.

There was an effect of brackish water depths on the MM content (Figure 4A), with an increasing linear effect at 180 and 450 DAP and a quadratic effect at 270 and 360 DAP (Figure 4B). Crude protein contents were quadratically influenced (*p* < 0.05) by brackish water depths at 270; 360; and 450 DAP (Figure 4B). There was no effect of brackish water depths (*p* > 0.05) on CP and EE contents at 180 DAP or on EE contents at 360 DAP (Figure 4B,C). However, there was a quadratic effect on EE contents at 270 and 450 DAP (Figure 4C).

**Figure 5:**
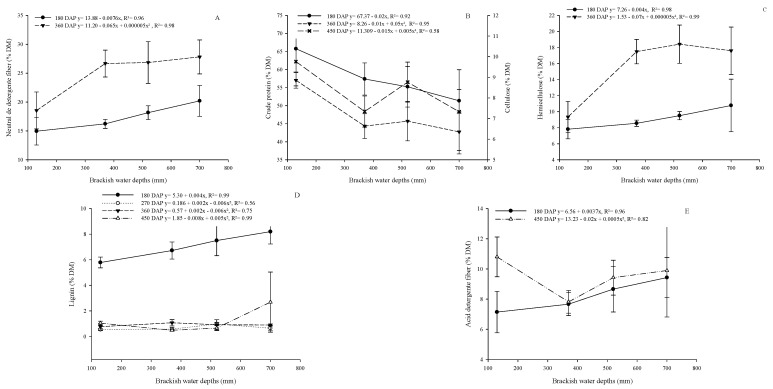
Effect of brackish water depths on the chemical composition of cactus pear under different levels of organic matter in a biosaline agriculture system. (Neutral detergent fiber (**A**), Non-fibrous carbohydrates (**B**), Hemicellulose (**C**), Acid detergent fiber (**D**), Lignin (**E**)). DM—dry matter.

The brackish water depth had an increasing linear (*p* < 0.05) influence on the NDF content at 180 DAP and a quadratic influence at 360 DAP (Figure 5A). Non-fibrous carbohydrates decreased linearly (*p* < 0.05) with increasing brackish water depths at 180 DAP and had a quadratic model fit at 360 DAP (Figure 5B). The cellulose of cactus pear showed a quadratic effect (*p* < 0.05) at 450 DAP as a function of applied brackish water depths (Figure 5B).

Brackish water depths influenced hemicellulose in a decreasing linear manner (*p* < 0.05) at 180 DAP and in a quadratic manner at 360 DAP (Figure 5C). ADF and LIG were influenced in an increasing linear manner (*p* < 0.05) at 180 DAP (Figure 5D,E), in a quadratic manner at 450 DAP for ADF (Figure 5D), and at 270; 360; and 450 DAP for lignin (Figure 5E).

**Figure 6:**
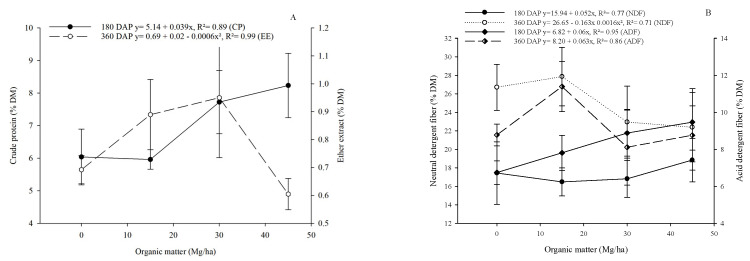

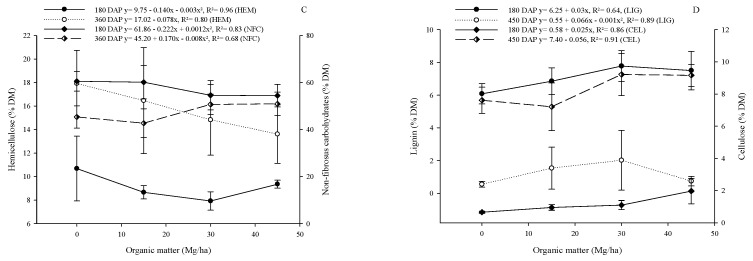
Effect of levels of organic matter on the chemical composition of cactus pear under different levels of organic matter in a biosaline agriculture system. (Crude protein and Ether extract (**A**), Neutral detergent fiber and Acid detergent fiber (**B**), Hemicellulose and Non-fibrous carbohydrates (**C**), Lignin and Cellulose (**D**)). FM—fresh matter, DM—dry matter.

The CP content increased as a function of organic matter (*p* < 0.05) at 180 DAP (Figure 6A). Hemicellulose and NFC showed a quadratic effect (*p* < 0.05) at 180 DAP according to the application of organic matter (Figure 6D). HEM and NFC were also influenced at 360 DAP, showing an increasing linear effect for HEM and a quadratic effect for NFC (Figure 6C). There was an increasing linear effect for LIG and CEL content (*p* < 0.05) at 180 DAP (Figure 6D). LIG was also influenced by organic matter (*p* < 0.05) in a quadratic manner at 450 DAP, and CEL at 450 DAP was influenced in an increasing linear manner (Figure 6D).

## 4. Discussion

Cactus pear responds to organic fertilization and irrigation with brackish water. Studies show that, when well irrigated and fertilized, *Opuntia* reaches yields of up to 47 t/ha/year [9]. Silva et al. [36] evaluated the rainfed cultivation system of the cactus pear for 745 days and found yields equivalent to 163.0 t/ha/year, which is similar to that of this study, in a shorter period of time (450 DAP) when using irrigation with brackish depths and organic fertilization. Lima et al. [37] observed no effect of irrigation depths with brackish water on the yields of green and dry matter of cactus pear, with averages of 114.5 and 7.3 t/ha/year, respectively (values close to those obtained here). As for cactus pear, cv. Gigante [38] argued that the application of 360 mm/year of C4S1 water was sufficient to increase the growth and yield of the crop. Nadaf et al. [39] investigated the irrigation of 13 cactus pear accessions with saline water (5.37 to 14.75 dS/m) and reported yields between 80 and 100 t/ha.

Morphological characteristics of cactus pear, such as plant height and width and cladode emergence, depend on the growth habit of clones and can influence crop productivity. According to Rocha et al. [40], *O. stricta* grows more laterally than vertically, which justifies the effect of brackish water depths only up to 360 DAP, since from this moment on, there is a greater limitation to plant growth due to the 1.6 m spacing between rows. At the beginning of the cycle (<180 DAP), the water demand by cactus pear is greatly reduced and the cladode emergence is low [41]. There are controversies about plant growth as a function of irrigation [42]; some studies show that there is no influence of the use of irrigation on growth [43], in contrast to [42], who observed that cactus responds well to irrigation.

The use of organic fertilization promoted a greater availability of nutrients; consequently, the plant presents higher elongation rates, resulting in taller or wider plants. Thus, the total number of cladodes of the plant reflects the probable stabilization or growth potential when it is still in a constant emergence stage [44,45].

Under water limitation, cactus pear significantly increases the number of lateral roots and, thus, water uptake, as explained by Fonseca et al. [10], who also found a reduction in water-use efficiency with increased water availability. The accumulation of water in the cladodes ensures it is better used by the plant and for a longer time. In rainfed conditions, Silva et al. [30] found the water-use efficiency of *O. stricta,* based on the green matter produced and the brackish water depth received by the crop, to be equal to 104.8 kg/ha/mm, being superior to the efficiency of clones of *Nopalea cochenillifera* (IPA Sertânia and Miúda), but not different from each other when water-use efficiency was based on dry matter (8.1 kg/ha/mm). When different organomineral and irrigated fertilizations were evaluated, *O. stricta* obtained WUEs of rainfall of 18.1 and 20.8 kg/ha/Mm in the second and third cropping cycles (cutting interval of 330 days), respectively [46].

The chemical composition values of cactus pear subjected to irrigation with brackish water exceed most of the values cited in the literature for rainfed crops. Araújo Filho et al. [47] analyzed different water regimes, and observed that they had no effect on nutritional quality, presenting average levels of MM, OM, NDF, ADF, CP, EE, and TC for the cactus pear of 17.08, 82.9, 16.66, 3.83, 3.82, 0.82, and 78.25%, respectively. Dubeux Jr. et al. [48] mentioned that the chemical composition of cactus pear can vary according to clone, water regime, age, order of emergence of cladodes, and fertilization, generally exhibiting 4 to 7% CP, 15 to 30% NDF, 18 to 20% ADF, and 50 to 55% NFC. In general, these results indicate that, regardless of brackish water depths and crop age, organic matter levels between 30 Mg/ha and 45 Mg/ha improved cactus performance. Rego et al. [49], on cactus pear cv. Miúda irrigated with a brackish water depth of 2.5 mm every 7 days, found DMP values at 360 DAP of 10.9; 15.4, and 10.8 Mg/ha/year when fertilized with 20, 40, and 60 Mg/ha of organic matter.

Values of DM, NDF (except at 180 DAP), HEM, and NFC (except at 360 DAP) reduced with increasing levels of organic matter, while the other variables (CP, ADF, LIG, and CEL) had their values increased. None of the analyzed variables were affected at all times of the crop cycle. Ramos et al. [50] studied the organic matter levels (0, 5, 10, 15, 20 ton/ha) in cactus pear cv. Gigante in rainfed conditions, and found values of 9.2% (DM), 14.2% (MM), 4.5% (CP), 1.7% (EE), 27.5% (NDF), 16.5% (ADF), 2.4% (LIG), 79.6% (TC), and 52.0% (NFC). Donato et al. [51] argued that the addition of 60 ton/ha/year in the soil increased the DM content of cactus pear at 600 days after planting from 7% (no manure) to 11%. Donato et al. [52] found a CP content of 9.5% in cactus pear cultivated without manure, and of 12% when 90 ton/ha/year of manure was added. Rocha et al. [40] studied the timing of harvests, and found increasing levels of DM (9.6%, 8.8%, 13.4%, and 12.1%) for *O. stricta* over the crop cycle (4, 8, 12, and 16 months).

## 5. Conclusions

The application of 304 mm/year of brackish water combined with an organic matter level of 30 to 45 Mg/ha offers an effective strategy for maximizing the productivity and quality of forage cactus, regardless of the harvest period, in biosaline production systems. Therefore, the minimal and seasonal use of brackish water is recommended, always in conjunction with organic fertilization based on the region’s rainfall.

## Figures and Tables

**Table 1:** Soil chemical composition in the experimental area.

Layer 0–20 cm												
EC	pH	C	N	P	K	Na	Ca	Mg	H + Al	SB	CEC	V
mS/cm	-	g/kg		mg/dm^3^	cmol/dm^3^							%
1.90	6.0	6.8	0.4	11.4	0.3	0.9	2.6	2.8	2.6	3.2	5.8	54

EC = electrical conductivity of the saturation extract; pH determined in water at a ratio of 1:2.5; C = total carbon; P = available phosphorus extracted by Mehlich; K = exchangeable potassium; Na = exchangeable sodium; Ca = exchangeable calcium; Mg = exchangeable magnesium; H + Al = potential acidity; SB = sum of bases; CEC = cation exchange capacity at pH 7.0; V = base saturation.

**Table 2:** Effect of brackish water depths on the growth and productivity of cactus pear under different levels of organic matter in a biosaline agriculture system.

Organic Matter	Brackish Water Depths (mm)				SEM	*p*-Valor
	130	370	520	700		
Plant height (cm)						
0	34.18 ± 5.06	37.31 ± 5.43	39.40 ± 5.11	42.87 ± 4.01	3.81	0.434
15	42.62 ± 7.83	46.18 ± 6.84	50.00 ± 6.36	53.00 ± 11.88	3.81	0.243
30	41.62 ± 8.66 b	48.31 ± 11.07 b	62.43 ± 15.62 a	69.81 ± 17.94 a	3.81	<0.001
45	41.31 ± 4.82 b	56.93 ± 9.82 a	58.50 ± 12.55 a	59.37 ± 13.44 a	3.81	0.002
Total number of cladodes (n°)						
0	7.75 ± 3.00	8.75 ± 1.92	6.18 ± 0.78	7.25 ± 2.00	1.15	0.463
15	9.87 ± 4.13	11.18 ± 2.14	10.37 ± 2.25	10.93 ± 4.53	1.15	0.854
30	11.06 ± 2.95 b	12.31 ± 1.73 b	13.62 ± 4.17 b	18.37 ± 5.70 a	1.15	<0.001
45	9.31 ± 2.06 b	11.06 ± 1.38 ab	13.27 ± 3.69 ab	14.56 ± 4.11 a	1.15	0.007
Fresh matter production (t/ha)						
0	19.32 ± 6.72	18.75 ± 3.69	17.70 ± 6.65	16.58 ± 5.41	12.48	1.002
15	20.92 ± 14.28	58.15 ± 25.81	48.63 ± 25.89	65.23 ± 36.48	12.48	0.066
30	18.27 ± 12.44 b	68.31 ± 29.39 a	81.91 ± 44.37 a	110.95 ± 53.34 a	12.48	<0.001
45	48.47 ± 14.95 b	111.87 ± 57.84 a	72.49 ± 39.47 ab	113.13 ± 45.18 a	12.48	<0.001
Dry matter productivity (t/ha)						
0	2.16 ± 0.94	1.75 ± 0.28	1.69 ± 0.64	1.46 ± 0.49	0.93	0.963
15	1.97 ± 1.15 b	4.64 ± 1.89 ab	4.19 ± 2.42 ab	5.86 ± 2.89 a	0.93	0.030
30	1.69 ± 1.06 b	5.38 ± 2.54 b	6.66 ± 3.87 ab	9.08 ± 3.69 a	0.93	<0.001
45	4.14 ± 1.02 b	7.99 ± 3.52 a	5.80 ± 2.78 ab	8.53 ± 3.34 a	0.93	<0.001
Water productivity (ton/ha)						
0	17.16 ± 5.78	16.99 ± 3.42	16.00 ± 6.02	15.11 ± 4.92	11.57	1.003
15	18.95 ± 13.22	53.50 ± 24.12	44.44 ± 23.47	59.37 ± 33.60	11.57	0.070
30	16.57 ± 11.38 b	62.93 ± 26.85 a	75.25 ± 40.50 a	101.86 ± 49.68 a	11.57	<0.001
45	44.33 ± 14.01 b	103.87 ± 54.34 a	66.69 ± 36.70 ab	104.60 ± 42.23 a	11.57	<0.001

Averages followed by equal letters on the same line do not differ by the Tukey test at 5% probability (*p* < 0.05).

## Data Availability

Data are contained within the article.
